# PLUNC Proteins Positivity in Patients with Chronic Rhinosinusitis: A Case-Control Study

**DOI:** 10.1155/2014/853583

**Published:** 2014-07-15

**Authors:** Desiderio Passali, Codrut Sarafoleanu, Claudiu Manea, Michele Loglisci, Francesco Maria Passali, Jacopo Cambi, Cristina Iosif, Eugenia Panaitescu, Luisa Maria Bellussi

**Affiliations:** ^1^Direttore Otorinolaringoiatria, Azienda Ospedaliera Universitaria Senese, Viale Bracci, 53100 Siena, Italy; ^2^ENT Department, Sfanta Maria Clinical Hospital, Carol Davila University of Medicine and Pharmacy 37, Dionisie Lupu Street, 020021 Bucharest, Romania; ^3^ENT Department, Univeristá degli Studi “Tor Vergata” di Roma, U.O.C. di Otorinolaringoiatria, Viale Oxford 81, 00133 Rome, Italy; ^4^Anatomopathology Department, Sfanta Maria Clinical Hospital, Carol Davila University of Medicine and Pharmacy 37, Dionisie Lupu Street, 020021 Bucharest, Romania; ^5^Department of Medical Informatics and Biostatistics, Sfanta Maria Clinical Hospital, Carol Davila University of Medicine and Pharmacy 37, Dionisie Lupu Street, 020021 Bucharest, Romania

## Abstract

*Introduction*. Innate immunity is the first protection against microorganisms. Nowadays, there is a growing interest in innate immune molecule known as palate, lung, nasal epithelial clone (PLUNC). PLUNC is a specific product of the airways, of approximately 25 kDa, encoded by adjacent genes found within a 300 kb region of chromosome 20; these proteins must be detected predominantly in the upper respiratory tract. *Materials and Methods*. We performed a case-control study to investigate the presence of this protein in nasal tissue of patients affected by chronic rhinosinusitis. 59 patients were enrolled (44 cases, 15 controls). We have examined the correlation between the presence of pathology and the PLUNC proteins positivity. *Results*. 100% of controls have a +++ rated PLUNC proteins positivity, while cases have a lower percentage of positivity. We used *χ* ^2^ statistical test to analyze the results of the study and there is a difference statistically significant between cases and controls in PLUNC proteins positivity. *Conclusions*. These observations suggest that, in response to agents or chemical factors, nasal mucosal epithelium will react and produce PLUNC proteins. So PLUNC proteins have a protective function on upper airways mucosa, as we can see by evaluating the high positivity in control group.

## 1. Introduction

In multicellular organisms, innate immunity is the first protection against microorganisms. The innate immune system consists of a group of polypeptides and proteins which work together to respond to threats from microbes and/or chemical agents. Such molecules are often produced in regions of the body that interface with the environment, where innate defense is a major requirement, such as upper and lower airways (nasal, tracheal, and bronchial tissues), major salivary glands and minor mucosal glands of the oral cavity, gastric mucosa, and the skin [1].

Nowadays, there is a growing interest in the proposed innate immune molecule known as palate, lung, nasal epithelial clone (PLUNC).

PLUNC genes are expressed predominantly in the upper respiratory tract, nasal mucosa, and oral cavity. PLUNC proteins are structurally similar to lipopolysaccharide binding protein (LBP) and bactericidal/permeability-increasing protein (BPI), and so authors hypothesized that PLUNC proteins may function in the innate immune defense of the respiratory tract, also acting as sensors of bacteria in airways and oral cavity.

PLUNC mRNA has been identified in upper airways mucosa and also the proteins were localized to the same sites too, while their expression is absent in small airways and peripheral lung.

PLUNC proteins are encoded by adjacent genes found within a 300 kb region of chromosome 20 [2], suggesting that they may be under transcriptional control of shared genomic elements, and expression data shows that these proteins are found in overlapping regions of the pulmonary, nasopharyngeal, and oral epithelium, sites where the bactericidal/permeability-increasing proteins, another kind of innate immune system protein, are not expressed.

PLUNC is a specific product of the upper and lower airways, of approximately 25 kDa, and is detected in oral and respiratory fluids and produced by upper airways mucosa including saliva and nasal lavage fluid. A prolonged exposure to airway irritants leads to decreased amounts of PLUNC in the upper airways, possibly because of a toxic effect on secretory cells. It is possible that PLUNC may be used as a marker of airway irritation and that decreased levels of PLUNC may promote inflammation in the nasal mucosa.

Among all the proteins present in human nasal lavage fluid, only PLUNC proteins were adsorbed onto a lipopolysaccharide-coated surface, which is central to host defenses against Gram-negative bacteria [3, 4].

Family members have been grouped (and named) based on their sizes. Short proteins comprise SPLUNC-1 (Short PLUNC-1; 256 amino acids), SPLUNC-2 (Short PLUNC-2; 249 amino acids), and SPLUNC-3 (Short PLUNC-3; 253 amino acids). Long proteins comprise LPLUNC-1 (Long PLUNC-1; 484 amino acids), LPLUNC-2 (Long PLUNC-2; 458 amino acids), LPLUNC-3 (Long PLUNC-3; 463 amino acids), and LPLUNC-4 (Long PLUNC-4; >469 amino acids).

The short proteins have homology only to the N-terminal domain of bactericidal/permeability-increasing protein, while the long proteins have homology to both the N- and C-terminal domains of bactericidal/permeability-increasing protein and LBP [5].

Primary protein sequence is quite similar to equine protein latherin. Latherin, originally isolated from horse sweat and subsequently shown to be present in saliva, exerts potent surfactant effects on air/liquid interfaces. Latherin is a member of the equine PLUNC cluster and shares an interesting property with the human PLUNC protein in that it exhibits an unusually high degree of hydrophobicity. Hydrophobic residues make up a similar proportion of the total amino acid content in latherin and PLUNC (44.2% in latherin, 44.7% in PLUNC) [6].

PLUNC proteins are also known as SPLUNC-1 (Short PLUNC-1), LUNX (lung-specific X protein), NASG (nasopharyngeal carcinoma-related protein), or SPURT (secretory protein in upper respiratory tracts).

About the association between altered PLUNC expression and airway inflammation, many authors have hypothesized that PLUNC may be an airway surfactant and so may have an antibiofilm activity too [7] interrupting biofilm formation by most common airway pathogens and furthermore can be upregulated after bulbectomy to prevent infection after injury [8].

The antimicrobial effect of PLUNC and its surfactant activity of lowering the surface tension of the airways mucosa may represent a novel form of innate immunity that limits bacterial colonization of the airways; a dysfunction of the innate immune system may play a permissive role in chronic rhinosinusitis pathogenesis, creating an environment that fosters increased microbial colonization [9].

PLUNC expression was significantly reduced in the mucosal epithelia and submucosal glands in the patients with multibacterial colonization, particularly those mediated by* Staphylococcus aureus* and* Pseudomonas aeruginosa*. These results suggest that patients affected by chronic rhinosinusitis with reduced PLUNC expression might have immune defect in defeating bacterial infection; thus reduced PLUNC expression might facilitate recurrent* Staphylococcus aureus* and* Pseudomonas aeruginosa* infections. So the low tissue levels of PLUNC expression could be considered as a mucosa inflammatory measurement to assess the inflammatory of sinus mucosa that might be due to* Staphylococcus aureus* and* Pseudomonas aeruginosa* colonization.

Numerous mechanisms underlie the interaction between PLUNC expression and inflammation in patients with microbial infections. PLUNC expression could be induced and upregulated by microbial infection. There is evidence that PLUNC is secreted by neutrophils upon bacterial stimulation [10]. PLUNC has been identified as an antimicrobial host defense peptide that may contribute to airway health through both bactericidal and nonbactericidal mechanisms, such as alleviating inflammation by reducing the production of IL-8, IL-6, and IL-1b 5-6 [11, 12].

So PLUNC might represent a novel predictive outcome biomarker for patients with chronic rhinosinusitis bacterial colonization. Continuous antibiotic use based on microbial culture reports, intensive nasal treatment, or sinus irrigation should be performed in patients with reduced PLUNC expression so as to decrease the possibility of chronic pathology.

The regulation of PLUNC protein has not been fully elucidated. Treating normal human nasal epithelial cells with IL-1b and TNF-a, which are the most important proinflammatory mediators presenting the airway, the levels of PLUNC gene expression and protein expression were not significantly altered. Moreover, PLUNC protein expression was not affected by various inflammatory states of the nasal tissue. These findings suggest that inflammatory mediators may not regulate the expression of PLUNC but suggest that PLUNC protein was secreted constantly [13].

We can hypothesize that reduction in the expression of PLUNC proteins may contribute to chronic rhinosinusitis pathogenesis not only due to reduction of its antimicrobial effects but also due to alterations of its physicochemical effects.

In fact, some current theories of chronic rhinosinusitis pathogenesis have focused not only on defects in the production of innate host defense proteins, barrier defects, and the role for superantigens, but also on mucociliary dysfunction.

PLUNC proteins have a role in suppressing the activation of epithelial sodium channels (ENaC), which has implications in mucociliary clearance [14]. The dysregulation of ENaC can cause an alteration of the mucociliary clearance and this fact can be considered another mechanism by which pathogens are able to cause chronic inflammation.

## 2. Materials and Methods

We performed a case-control study to investigate the presence of this protein in the nasal tissues of patients affected by chronic rhinosinusitis.

Rhinosinusitis is defined as inflammation of the nose and the paranasal sinuses characterized by two or more symptoms, one of which should be either nasal blockage/obstruction/congestion or nasal discharge (anterior/posterior nasal drip), with or without facial pain/pressure, with or without reduction or loss of smell, and either endoscopic signs of polyps and/or mucopurulent discharge primarily from middle meatus and/or oedema/mucosal obstruction primarily in middle meatus and/or mucosal changes within the ostiomeatal complex and/or sinuses at CT scan [15].

We enrolled 44 patients with chronic rhinosinusitis while the control group is represented by 15.

Among the patients, 28 were male and 16 were female. The ages ranged between 21 and 71 years.

Control subjects were 9 male and 6 female, aged between 24 and 63 years.

Furthermore, we grouped the patients according to the presence of allergy, asthma, and smoking habits. Nine patients (6 male, 3 female) were allergy sufferers, 8 patients (4 male, 4 female) had asthma, and 4 patients (3 male, 1 female) were smokers. Skin prick tests including both pollen and perennial allergens were performed to evaluate the presence of allergy while asthma was diagnosed by a pneumologist based on the evaluation of airway responsiveness. All the smoker subjects had moderate smoking habits (from 10 to 20 cigarettes a day). Tissues were taken from the ethmoid sinus of cases while normal mucosal tissues were obtained from the turbinate mucosa for the 15 controls.

We focused on the most highly expressed PLUNC family member, SPLUNC-1, to elucidate its expression in chronic rhinosinusitis. All the samples were paraffin-embedded. To ascertain the intensity of PLUNC protein expression [16], we deparaffinized, dehydrated with graded alcohol series, and rehydrated them. All samples were then colored with streptavidin-peroxidase method. After that, they were blocked for 30 minutes with 0.3% H_2_O_2 _in methanol and incubated with serum for 1 hour. The sections were then incubated for 30 minutes with 0.1 mL of goat polyclonal antibody against PLUNC protein (1 : 200 dilution; R&D Systems, Minneapolis), followed by a Rabbit Anti-Goat IgG HRP Affinity Purified Pab (R&D Systems, Minneapolis) for another 30 minutes.

The sections were then colorized with diaminobenzidine/hydrogen peroxide and counterstained with hematoxylin. Finally, the sections were examined under a microscope (40–100x).

The PLUNC proteins positivity was classified as +++ (>50% positive cells), ++ (26 to 50% positive cells), + (≤25% positive cells), or − (no positive cells).

We evaluated the correlation between patients and control subjects for frequencies of PLUNC proteins positivity using the *χ* ^2^ test with a statistical significance level of 95% (*P* value <0.05).

## 3. Results

In the present study, we focused our attention on the most expressed PLUNC family molecules in sinus and nasal tissues, SPLUNC-1. We were able to confirm reduced levels in the nasal tissues of patients affected by chronic rhinosinusitis due to a decreased number of glands.

**Figure 1:**
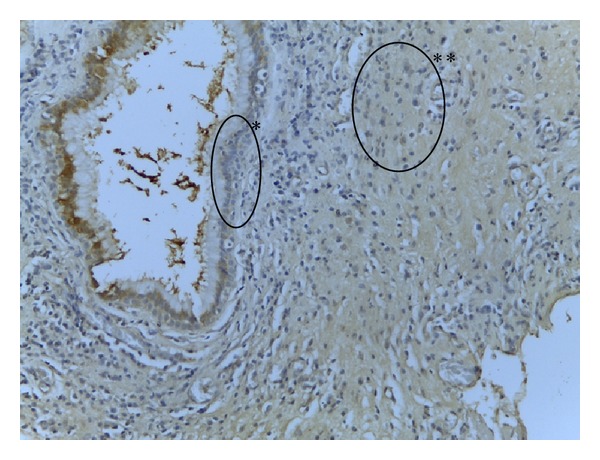
Nasal mucosa: PLUNC positive (+) IHC 40x. Very weak positivity for PLUNC antibodies in epithelium (∗) and small lymphocytes (∗∗).

**Figure 2:**
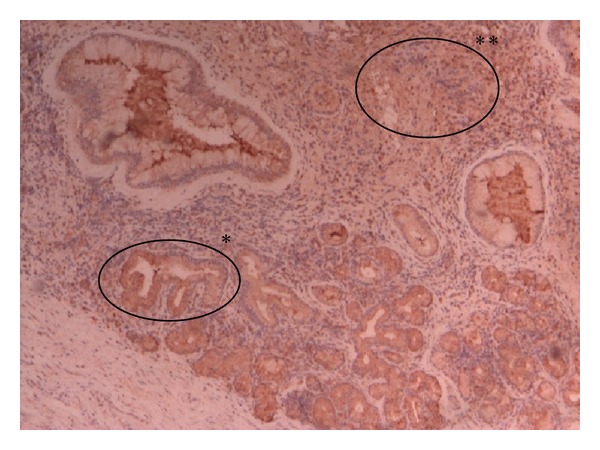
Nasal mucosa: PLUNC positive (++) IHC 40x. ++ PLUNC antibodies positivity in epithelium (∗) and in stroma (∗∗).

**Figure 3:**
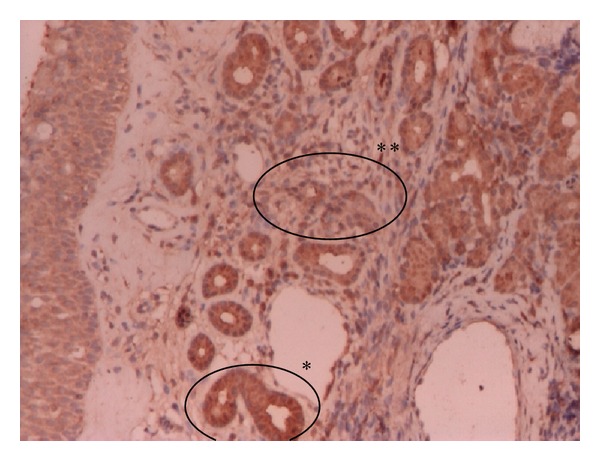
Middle turbinate mucosa: PLUNC positive (+++) IHC 100x. +++ PLUNC antibodies positivity in epithelium (∗) and in stroma (∗∗).

From the data collected, in fact, we can see that all the enrolled in control group have a +++ rated positivity (such as in Figures 1, 2, and 3), while the patients affected by rhinosinusitis have a significant lower PLUNC proteins positivity.

**Figure 4:**
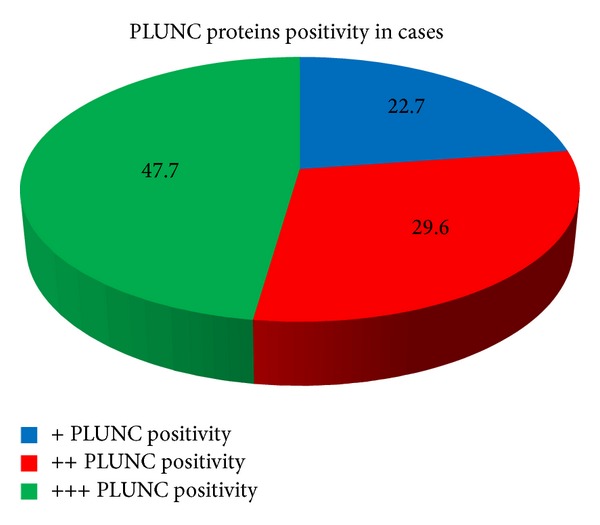
Graphical representation of PLUNC proteins positivity in cases.

Only about half of the case patients have a +++ rated positivity (Table 1 and Figure 4), while the other half have a ++ or + rated positivity (nobody, both cases and controls considered, has a − rated expression).

We examined the correlation between the presence of pathology and the PLUNC proteins positivity using *χ* ^2^ statistical test to analyze the results of the study.

The differences in PLUNC proteins positivity between patients and control group are statistically significant (*χ* ^2^ test, *P* value =0.0016).

Conversely, in our cases, there was not a statistically significant correlation between PLUNC proteins positivity and the presence of allergy, asthma, and smoking habits, even if according to other series of data there may be a correlation between PLUNC expression, allergy, and smoking habits [17].

## 4. Conclusions

In our study of 59 patients, statistical analysis indicated that the amount of PLUNC proteins expression is significantly correlated with the presence of chronic rhinosinusitis.

These observations suggest that, in response to irritation by infectious agents or chemical factors, the nasal mucosal epithelium [13, 18] will react and produce anti-inflammatory agents, including PLUNC proteins, as a defense against the irritant agents.

On irritation of the respiratory mucosa by infectious agents or chemical factors, production of such anti-inflammatory agents is upregulated as a defense mechanism.

In cases of defects or destruction of the immune defense function of the respiratory mucosa, inflammation would fall into a vicious circle, and diseases based on infection and/or inflammation may arise.

The decrease in the expression of these molecules is probably explained by a decrease in the number of glands in pathological tissues and so by a decreased ability to clear pathogens.

These defects of defense may contribute to the chronic inflammatory response and so the pathogenesis of chronic rhinosinusitis could be explained.

## Figures and Tables

**Table 1:** PLUNC proteins positivity in cases and controls.

			Patients	Control group	Total
PLUNC positivity	**+**		10	0	10
		%	22,7%	0%	17%
	**++**		13	0	13
		%	29,6%	0%	22%
	**+++**		21	15	36
		%	47,7%	100,0%	61%
	Total		**44**	**15**	**59**
		%	**100,0%**	**100,0%**	**100,0%**
